# Enhancing anti-tumor potential: low-intensity vibration suppresses osteosarcoma progression and augments MSCs' tumor-suppressive abilities

**DOI:** 10.7150/thno.90945

**Published:** 2024-01-27

**Authors:** Xue Xiong, Qingji Huo, Kexin Li, Changpeng Cui, Chunyi Chang, Charles Park, BonHeon Ku, Chin-Suk Hong, HeeChang Lim, Pankita H. Pandya, M. Reza Saadatzadeh, Khadijeh Bijangi-Vishehsaraei, Chien-Chi Lin, Melissa A. Kacena, Karen E. Pollok, Andy Chen, Jing Liu, William R. Thompson, Xue-Lian Li, Bai-Yan Li, Hiroki Yokota

**Affiliations:** 1 Department of Pharmacology, School of Pharmacy, Harbin Medical University, Harbin 150081, China.; 2 Department of Biomedical Engineering, Indiana University Purdue University Indianapolis, Indianapolis, IN 46202, USA.; 3 Weldon School of Biomedical Engineering, Purdue University, West Lafayette, IN 47907, USA.; 4 Department of Physics, Indiana University Purdue University Indianapolis, Indianapolis, IN 46202, USA.; 5 Department of Mechanical Engineering, Pusan National University, Busan 46241, Korea.; 6 Department of Mechanical Engineering, Ulsan College, Ulsan 44022, Korea.; 7 Indiana University Simon Comprehensive Cancer Center, Indiana University School of Medicine; Indianapolis, IN 46202, USA.; 8 Department of Pediatrics, Indiana University School of Medicine; Indianapolis, IN 46202, USA.; 9 Department of Pediatric Hematology and Oncology, Indiana University School of Medicine; Indianapolis, IN 46202, USA.; 10 Department of Orthopaedic Surgery, Indiana University School of Medicine; Indianapolis, IN 46202, USA.; 11 Indiana Center for Musculoskeletal Health, Indiana University School of Medicine; Indianapolis, IN 46202, USA.; 12 Department of Medical and Molecular Genetics, Indiana University School of Medicine, Indianapolis, IN 46202 USA.; 13 Department of Physical Therapy, Indiana University, Indianapolis, IN 46202, USA.

**Keywords:** vibration, osteosarcoma, MSCs, iTSCs, conditioned medium, glycolytic enzymes

## Abstract

**Rationale:** Osteosarcoma (OS), a common malignant bone tumor, calls for the investigation of novel treatment strategies. Low-intensity vibration (LIV) presents itself as a promising option, given its potential to enhance bone health and decrease cancer susceptibility. This research delves into the effects of LIV on OS cells and mesenchymal stem cells (MSCs), with a primary focus on generating induced tumor-suppressing cells (iTSCs) and tumor-suppressive conditioned medium (CM).

**Methods:** To ascertain the influence of vibration frequency, we employed numerical simulations and conducted experiments to determine the most effective LIV conditions. Subsequently, we generated iTSCs and CM through LIV exposure and assessed the impact of CM on OS cells. We also explored the underlying mechanisms of the tumor-suppressive effects of LIV-treated MSC CM, with a specific focus on vinculin (VCL). We employed cytokine array, RNA sequencing, and Western blot techniques to investigate alterations in cytokine profiles, transcriptomes, and tumor suppressor proteins.

**Results:** Numerical simulations validated LIV frequencies within the 10-100 Hz range. LIV induced notable morphological changes in OS cells and MSCs, confirming its dual role in inhibiting OS cell progression and promoting MSC conversion into iTSCs. Upregulated VCL expression enhanced MSC responsiveness to LIV, significantly bolstering CM's efficacy. Notably, we identified tumor suppressor proteins in LIV-treated CM, including procollagen C endopeptidase enhancer (PCOLCE), histone H4 (H4), peptidylprolyl isomerase B (PPIB), and aldolase A (ALDOA). Consistently, cytokine levels decreased significantly in LIV-treated mouse femurs, and oncogenic transcript levels were downregulated in LIV-treated OS cells. Moreover, our study demonstrated that combining LIV-treated MSC CM with chemotherapy drugs yielded additive anti-tumor effects.

**Conclusions:** LIV effectively impeded the progression of OS cells and facilitated the transformation of MSCs into iTSCs. Notably, iTSC-derived CM demonstrated robust anti-tumor properties and the augmentation of MSC responsiveness to LIV via VCL. Furthermore, the enrichment of tumor suppressor proteins within LIV-treated MSC CM and the reduction of cytokines within LIV-treated isolated bone underscore the pivotal tumor-suppressive role of LIV within the bone tumor microenvironment.

## Introduction

OS is a malignant bone tumor originating from mesenchymal cells, constituting the most prevalent form of primary bone cancer 1. Although it can manifest in any bone throughout the body, it predominantly afflicts the long bones, particularly the tibia and femur, of adolescents and young adults 2, 3. Treatment for OS primarily involves surgical intervention and chemotherapy. The standard chemotherapeutic regimen, known as MAP therapy, encompasses methotrexate, doxorubicin, and cisplatin 4. Notably, metastasis most commonly occurs within the lung parenchyma, significantly impacting clinical outcomes in a negative manner 5. Presently, the effectiveness of targeted medications remains limited due to the marked heterogeneity of OS 6. Consequently, developing a safe and efficacious treatment for OS is a critical necessity.

The human bone is a vibrant and dynamic tissue that reacts to mechanical stimuli, playing a pivotal role in maintaining equilibrium 7. Beyond upholding bone health, exercise contributes to diminishing cancer susceptibility 8, despite its demanding nature for individuals with cancer. Offering a substitute for physical exertion, LIV presents motion-like mechanical stimuli 9. Studies have indicated that subjecting MSCs to LIV prompts a shift in their developmental trajectory, favoring osteoblasts over adipogenic cells 10. A growing body of evidence demonstrates the potential of LIV to enhance bone mineral density and mitigate the risk of bone fractures 11-13. Furthermore, LIV's influence extends to cancer cells, altering their biophysical traits. This includes the reinforcement of plasma membrane rigidity and the inhibition of invasive tendencies in breast cancer cells 14. In this study, our primary focus was to explore the impact of LIV on cell biomechanics and morphology. Additionally, we delved into the examination of tumorigenic signaling and behaviors, specifically emphasizing the intricate interplay between OS cells and MSCs.

A novel methodology has been devised to generate iTSCs and cultivate tumor-suppressive CM. In a paradoxical twist, MSCs were reprogrammed into iTSCs through the overexpression of oncogenes such as β-catenin in the Wnt signaling pathway, Akt in the PI3K signaling pathway, and cMyc 15, 16. Additionally, the creation of iTSCs was achieved via the administration of synthetic agents, including BML284 and YS49, serving respectively as activators of the Wnt and PI3K pathways 15, 17. Intriguingly, within the CM derived from iTSCs, a collection of tumor-suppressing proteins was identified. This ensemble comprised PCOLCE, H4, PPIB, and ALDOA, proteins hitherto unreported for their tumor-inhibitory properties 17. The present investigation is centered on exploring the capacity of LIV to affect the conversion of MSCs into iTSCs.

VCL, a protein closely associated with focal adhesions, is prominently present at sites of cell-cell and cell-surface interactions 18, 19. Notable proteins like talin, IpaA, β-catenin, α-catenin, and α-actinin interact with its head region 20, while the tail region binds with paxillin, acidic phospholipids, and actin 21. The mounting body of evidence underscores the role of VCL in mediating the transmission of mechanical forces from the extracellular matrix to the cytoskeleton, primarily due to its robust link with the actin cytoskeleton 22, 23. The extent of VCL recruitment in focal adhesions correlates directly with the magnitude of the force exerted on the substrate; cells lacking VCL or expressing its mutant variants experience compromised mechanosensing capabilities 24. This study delves into examining VCL's influence on modulating the tumor-suppressive functionality of iTSCs originating from MSCs treated with LIV.

ALDOA, a constituent of the glycolytic enzyme family responsible for generating ATP molecules from glucose, has garnered significant attention 25, 26. While multiple studies point towards ALDOA's role in promoting tumorigenesis across various cancer types 27, 28. Our findings paradoxically reveal ALDOA's enrichment in CM derived from iTSCs. Remarkably, ALDOA emerges as an extracellular protein contributing to the suppression of tumor progression. This unconventional extracellular role of ALDOA mirrors the dual nature of Moesin, which serves as both a tumor suppressor and promoter in its extracellular and intracellular domains, respectively 15. Of particular intrigue, a peptide fragment of ALDOA denoted as P04 (IGEHTPSALAIMENANVLAR) exhibits anti-tumor properties, akin to another antitumor peptide. In the current study, our focus revolves around investigating the potential elevation of ALDOA expression within CM derived from MSCs treated with LIV. Additionally, we seek to explore the application of P04 as a strategy to impede the progression of OS.

Despite the widespread use of LIV in the frequency range of 10 - 100 Hz and with a magnitude of 0.1 to 1 gravitational acceleration (G) for assessing cellular and molecular reactions, a comprehensive biomechanical investigation of the effects of LIV on treated cells remains incomplete. Here, we conducted modal analysis and predicted natural frequencies using simplified cell models with different mechanical properties and shapes. By combining numerical predictions with subsequent experimental verification, we selected a frequency range of 30 - 90 Hz with gravity magnitudes from 0.3 - 1 G.

## Materials and Methods

### Cell culture and agents

Human OS cell lines, namely MG63 and U2OS (86051601-1VL and 92022711-1VL, Sigma, St. Louis, MO, USA), were included in this study. Additionally, a patient-derived xenograft (PDX) TT2-77 OS xenoline, murine K7M2 OS cell line (ATCC, Manassas, VA, USA), RAW264.7 pre-osteoclast cells (ATCC), and murine MSCs isolated from the bone marrow of the C57BL/6 strain, were also employed. These cell lines and xenoline were all cultured in DMEM. Human MSCs (PT-2501, Lonza, Basel, Switzerland) were grown in MSCBM (PT-3001, Lonza). The culture medium used was supplemented with 10% fetal bovine serum and antibiotics (100 units/mL penicillin, and 100 µg/mL streptomycin; Life Technologies, Grand Island, NY, USA). All cells were maintained under standard conditions at 37°C with a 5% CO_2_ atmosphere.

### Application of LIV

LIV was administered using a specially designed vibration apparatus. Cells within a culture dish (35 × 10 mm) underwent horizontal (h-LIV), 30˚ angle, 60˚ angle, and v-LIV at frequencies of 60 or 90 Hz. These vibrations were applied at intensities of 0.1, 0.3, 0.5, 0.7 and 1 g. In the case of MTT, EdU, scratch, and transwell assays, cells were subjected to two sessions of 20-min LIV exposure, each separated by a 3-h interval (Figure S1). To establish a reference, a control group was maintained under identical growth conditions. These control samples were placed on the LIV device but were not exposed to any vibrations.

### Cellular imaging

Around 100 cells were placed into each well of a 96-well plate. Once the cells adhered to the plate surface, we examined their morphological transformations prior to and following the application of LIV (oscillatory vibrations) at a frequency of 90 Hz and an acceleration of 0.7 g. To document these changes, we utilized the Cellcyte X Live Cell Analyzer (Echo, San Diego, CA, USA) in combination with light microscopy. Subsequently, we employed Image J to quantify the contact area of these cells.

### Hydrogel coating preparation

To make substrates with various stiffnesses, we made gelatin-based hydrogels according to our previous method 29. Briefly, gelatin was modified with norbornene (NB) and carbohydrazide (CH) moieties, yielding GelNB-CH for crosslinking with PEG-tetra-thiol (PEG4SH) to form thiol-norbornene click hydrogels. The hydrogels were either used as prepared or dynamically stiffened with oxidized dextran (oDex) by forming a secondary hydrazone bonding network in the primary thiol-norbornene hydrogels.

### MTT and EdU assay

MTT-based assessment of metabolic activity involved the use of approximately 2,000 cells within 96-well plates (Corning, Glendale, Arizona, USA). On the second day, CM was introduced, and on the fourth day, cells were subjected to staining with 0.5 mg/ml thiazolyl blue tetrazolium bromide (#M5655, Sigma). Metabolic activities were quantified by measuring the optical density at 570 nm. In the EdU assay, around 1,000 cells were seeded in 96-well plates on the first day. On the second day, CM was added, and cellular proliferation was assessed using a fluorescence-based cell proliferation kit (Click-iT™ EdU Alexa Fluor™ 488 Imaging Kit; Thermo Fisher Scientific, Waltham, MA, USA) on the fourth day. Following fluorescent labeling, the count of fluorescently labeled cells was conducted, and the ratio of these labeled cells to the total cell count was calculated.

### 2D motility scratch assay and Transwell invasion assay

A 2D motility scratch assay was executed to assess two-dimensional migratory behavior. Roughly 4×10^5^ cells were seeded into 12-well plates. Upon cell attachment, a plastic pipette tip was utilized to generate a scratch within the cell layer. The suspended cells were eliminated, and CM was introduced. Images capturing the cell-free scratch zone were captured at time point 0 h. Subsequently, the newly occupied areas with cells were evaluated 24-h post-scratching. These areas were quantified using Image J (National Institutes of Health, Bethesda, MD, USA). In the context of a transwell invasion assay, approximately 5×10^4^ cells were suspended in 200 µL of serum-free DMEM and placed within the upper transwell chamber (Thermo Fisher Scientific) containing Matrigel (100 µg/mL). The lower chamber was supplemented with 800 µL of CM. After 48 h, cells that had penetrated the underside of the membrane were subjected to staining with Crystal Violet. A minimum of five randomly selected images were captured, and the average count of stained cells was subsequently determined.

### Osteoclast differentiation assay

The differentiation assay for RAW264.7 pre-osteoclasts was conducted within a 12-well plate setting. For 6 days, the pre-osteoclast cells were incubated in a medium containing 40 ng/mL of RANKL. During this incubation period, the culture medium was replaced once on the fourth day. Adherent cells were then fixed and subjected to staining using a tartrate-resistant acid phosphate (TRAP)-staining kit sourced from Sigma, following the guidelines provided by the manufacturer. The identification of mature osteoclasts involved the recognition of TRAP-positive multinucleated cells possessing more than three nuclei.

### Western blot analysis

Cultured cells were lysed using a radio-immunoprecipitation assay buffer, after which proteins were separated via 10-15% SDS gels and subsequently transferred onto polyvinylidene difluoride transfer membranes (Millipore, Billerica, MA, USA). Following this, the membrane underwent an overnight incubation with primary antibodies, followed by treatment with secondary antibodies conjugated with horseradish peroxidase (Cell Signaling, Danvers, MA, USA). Antibodies against VCL, Snail, p-Src, Src, CXCL1, CXCL2, PCOLCE, H4, PPIB, ALDOA, APP, ALP, Col Ι, cleaved caspase 3, caspase 3 (Cell Signaling), and OPN (Santa Cruz Biotechnology) were employed, with β-actin (Sigma) serving as a control. Quantification of protein levels was achieved using the SuperSignal West Femto Maximum Sensitivity Substrate (Thermo Fisher Scientific). Signal intensities were then quantified using a luminescent image analyzer (LAS-3000, Fuji Film, Tokyo, Japan).

### *Ex vivo* bone assay

A pair of femora were harvested from C57BL/6 female mice (approximately 10 weeks old), and connective tissues surrounding the bones were carefully removed. The femora were bisected at the mid-diaphysis, and a perforation was created on the opposite side using a 25-gauge needle. K7M2 OS cells (2.5 × 10^5^ cells) were suspended in 10 μL of culture medium and subsequently injected into the bone marrow cavity through the exposed end of the diaphysis. Subsequently, the bone specimens were placed within a petri dish and cultivated in 2 mL of DMEM supplemented with 10% FBS and 1% antibiotics. The cultivation was conducted at a temperature of 37 °C under an atmosphere of 5% CO_2_. To maintain optimal conditions, half of the culture medium was replaced daily with fresh medium. Following a period of daily vibrations for 2 weeks, the bone samples were utilized for Western blot analysis.

### Animal model

The animal procedures were approved by the Indiana University Animal Care and Use Committee (SC345R) and complied with the Guiding Principles in the Care and Use of Animals endorsed by the American Physiological Society. Mice were randomly housed five per cage and provided with mouse chow and water ad libitum. In the mouse model of osteolysis, 8-week-old C57BL/6 female mice (5 mice in the placebo and CM control groups, and 6 mice in the LIV group) received K7M2 OS cells (2.5×10^5^ cells in 20 µl PBS), into the tibia as an intra-tibial injection. From day 2, 50 µL of CM was injected intravenously from the tail vein after condensing 10 times with a filter (3 kDa cutoff). The animals were sacrificed on day 14, and tibiae were harvested.

### X-ray imaging and microCT imaging

Whole-body X-ray imaging was performed with a Parameter cabinet X-ray system (Kubtec, Stratford, CT, USA). Using the procedure previously described 30, tibial integrity was scored in a blinded manner on a scale of 0 to 3: 0 = normal with no indication of a tumor, 1 = clear bone boundary with slight periosteal proliferation, 2 = bone damage and moderate periosteal proliferation, and 3 = severe bone erosion. Micro-computed tomography (microCT) was also performed with Skyscan 1272 (Bruker-MicroCT, Kontich, Belgium). Using manufacturer-provided software, scans were performed at pixel size 10 μm and the images were reconstructed (nRecon v1.6.9.18) and analyzed (CTan v1.13). Using µCT images, trabecular bone parameters such as bone volume ratio (BV/TV), bone mineral density (BMD), and trabecular number (Tb.n) were determined in a blinded fashion.

### Plasmid transfection, RNA interference, and cytokine array

The overexpression of VCL was accomplished through the transfection of its plasmids (#58198; Addgene, Cambridge, MA, USA), while blank plasmids (FLAG-HA-pcDNA3.1; Addgene) served as the control. Employing the procedure outlined in a previous study 31, RNA interference was carried out using siRNA specifically targeting VCL (#AM16708; Thermo Fisher), accompanied by a negative siRNA (Silencer Select #1, Thermo Fisher) as a nonspecific control. Additionally, we employed a mouse XL cytokine array (R&D Systems) to assess the levels of 111 cytokines and chemokines in ex vivo bone cultures, both with and without LIV application.

### RNA sequencing analysis

TT2 cells were cultivated in a 35 × 10 mm dish, both with and without vibration-induced localized oscillatory vibrations (v-LIV), operating at a frequency of 90 Hz and an acceleration of 0.7 g. This regimen consisted of two 20-min sessions per day, spaced 3 h apart, over 3 days. The experimental design encompassed a control group (C1, C2, C3) and a v-LIV treated group (V1, V2, V3), each containing 3 samples. Total RNA extraction was promptly performed using an RNA extraction kit (QIAGEN, Germantown, Maryland, USA) within an hour following the final v-LIV session on the third day. Subsequently, polyadenylated RNA sequencing was executed at the Center for Medical Genomics at Indiana University School of Medicine.

### Numerical simulation

The finite element analysis, incorporating structure-acoustic coupling, was carried out using the COMSOL Multiphysics software (version 6.1, Altsoft, Woodstock, GA, USA). The analysis included vital constituents such as the cell membrane, cytoplasm, nucleus, and outer fluid. It is assumed that the nucleus maintains solidity, while the cytoplasm and outer fluid possess respective densities of 1,080 kg/m³ and a speed of sound of 1,500 m/s 32. To achieve a comprehensive grasp of cell dynamics, modal analysis was performed, thereby facilitating the extraction of natural frequencies and modes. These characteristics not only capture variations in cell shape but also encompass responses to external vibrations that resonate with these intrinsic frequencies. The focal point of the exploration lies in the computation and analysis of the initial five natural vibration modes. Furthermore, the study delved into the influence of variations in Young's modulus on these natural vibrations. The analysis also took a closer look at the impact of the initial cell shape - whether it is circular or elliptical - as determined by the aspect ratio along the major axis 33.

### Statistical analysis

For cell-based experiments, three or four independent experiments were conducted, and data were expressed as mean ± S.D. Statistical significance was evaluated using a one-way analysis of variance (ANOVA). Post hoc statistical comparisons with control groups were performed using Bonferroni correction with statistical significance at p < 0.05. The single and double asterisks in the figures indicate p < 0.05 and p < 0.01, respectively.

## Results

### Modal analysis of a cell in response to vertical vibrations

**Figure 1:**
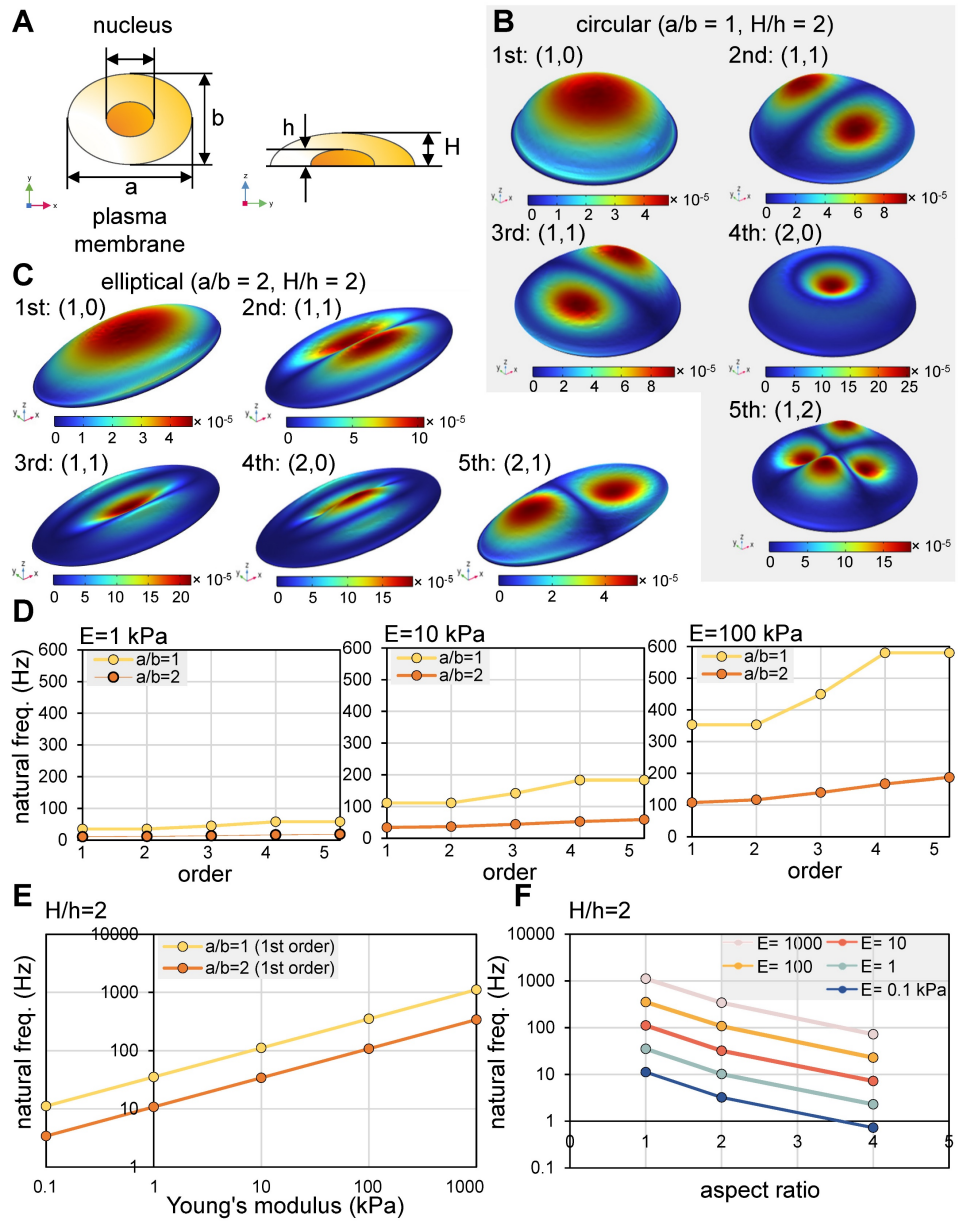
** Modal analysis of a cell in response to vertical vibrations.** (A) A single-cell model. Of note, a = major axis, b = minor axis, a/b = aspect ratio, h = height of a nucleus, and H = height of a plasma membrane. For the circular model (a/b = 1), the parameters were a = 30 μm, b = 30 μm, and H = 10 μm. For the elliptical model (a/b = 2), the parameters were a = 60 μm, b = 30 μm, and H = 10 μm. (B) The first five eigenmode shapes of a circular cell. (C) The first five eigenmode shapes for an elliptical cell. (D) The first five natural frequencies of an elliptical cell with Young's modulus of 1, 10, and 100 kPa with a/b = 1 and 2. (E) The first natural frequencies as a function of Young's modulus. (F) The first natural frequencies as a function of aspect ratio (a/b).

We conducted a modal analysis utilizing circular and elliptical cell models in response to vertical vibrations. The first five mode shapes are illustrated (Figure 1A-C), in which (n, m) represent the number of nodal circles and nodal lines 34. For both circular and elliptical cells, the initial four mode shapes present the sequential pattern of (1, 0), (1, 1), (1, 1), and (2, 0). Notably, the second and third modes exhibited (1, 1) configurations, displaying symmetrical shapes that are mutually perpendicular. In the case of circular cells, the natural frequencies of the second and third modes were found to be identical. Conversely, the fifth mode manifested as (1, 2) for circular cells, while it took the form of (2, 1) for elliptical cells.

The intrinsic frequencies were found to be influenced by both the rigidity and shape of the cells. For cells characterized by soft (Young's modulus, E = 1 kPa), intermediate (E = 10 kPa), and rigid (E = 100 kPa) properties, we derived estimations for the first five intrinsic frequencies, as depicted (Figure 1D) 34. Additionally, we graphically represented the impact of cell rigidity on the initial intrinsic frequency for both circular cells (aspect ratio, a/b = 1) and elliptical cells (a/b = 2) (Figure 1E). Intriguingly, the relationship between the intrinsic frequency and Young's modulus displayed a near-linear trend when plotted logarithmically. In terms of the aspect ratio's influence, the first intrinsic frequency exhibited a decrease with higher aspect ratios, corresponding to larger cell sizes (Figure 1F). In summation, our modal analysis suggests that vibrational frequencies within the range of 10 - 100 Hz possess the potential to effectively administer mechanical stimuli to cultured cells. However, the optimal frequency for efficacy is contingent upon the interplay of cell rigidity and shape.

### Alterations in OS cell shape in response to LIV

**Figure 2:**
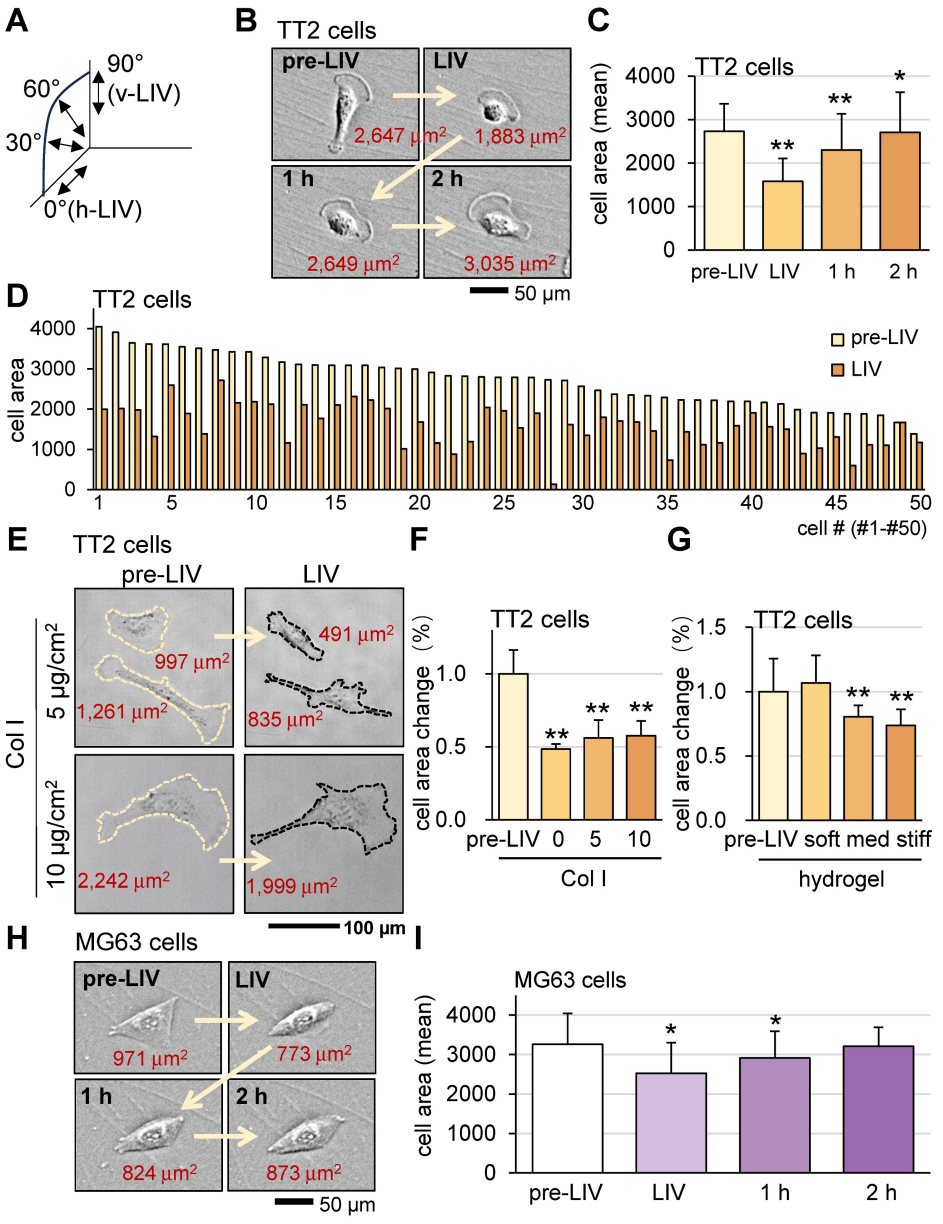
**Alterations in OS cell shape in response to LIV.** LIV was applied vertically (v-LIV) for 20 min at 90 Hz with a level of 0.7 g. The single and double asterisks indicate p < 0.05 and 0.01, respectively. (A) LIV direction, including v-LIV, 60˚, 30˚, and h-LIV. (B&C) Transient shrinkage of TT2 OS cells in response to v-LIV. (D) Reduction of the surface area for 50 TT2 cells by v-LIV. (E&F) Effects of type I collagen coating on the shrinkage of TT2 OS cells in response to v-LIV. (G) Shrinkage of TT2 OS cells in soft, medium, and stiff hydrogels in response to v-LIV. Of note, storage moduli G' is 1.2 kPa (soft), 4.3 kPa (med), and 7.6 kPa (stiff). (H&I) Transient shrinkage of MG63 OS cells in response to v-LIV.

To evaluate the responses of OS cells and MSCs to vibrations, this study mainly employed vertical low-intensity vibrations (v-LIV), but three other forms of vibrations including 60˚, 30˚, and horizontal low-intensity vibrations (h-LIV) were also used (Figure S2 and Figure 2A).

First, we applied v-LIV to OS cells (TT2 and MG63) and monitored cell shape and surface-contact area before and after the application of 20-min LIV at 90 Hz. The results showed that LIV significantly reduced the surface contact area of TT2 and MG63 cells (Figure 2B-F). The observed alteration is potentially affected by various cell-culture conditions. To evaluate the effect of surface adhesion and a three-dimensional environment, cells were incubated either on a type Ι collagen (Col Ι) coated surface or in soft, medium, and stiff hydrogels. The result showed that collagen coating and hydrogel stiffness affected the sensitivity to LIV (Figure 2G-I). A weak surface adhesion and stiff hydrogel made OS cells more sensitive to LIV.

### Effect of LIV on the viability and motility of OS cells

**Figure 3:**
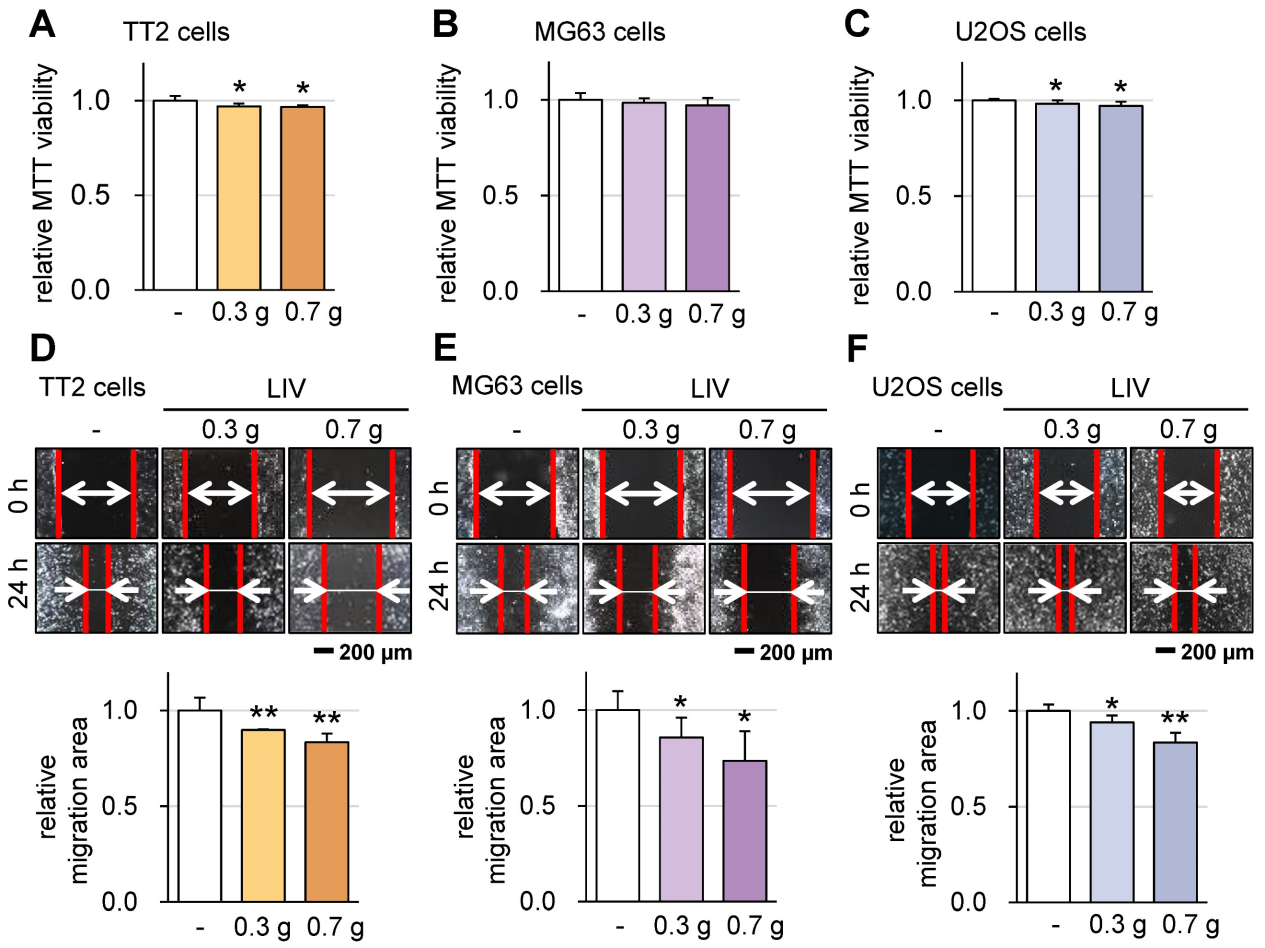
** Effect of LIV on the viability and motility of OS cells.** LIV was applied vertically (v-LIV) at 90 Hz with a level of 0.3 and 0.7 g. The single and double asterisks indicate p < 0.05 and 0.01, respectively. (A-C) MTT-based viability of OS cells (TT2, MG63, and U2OS) in response to LIV. (D-F) Reduction in scratch-based motility of OS cells (TT2, MG63, and U2OS) in response to LIV.

Subsequently, we investigated the impact of v-LIV on the tumorigenic behaviors of various OS cell lines, including TT2, MG63, and U2OS. Upon applying v-LIV at frequencies of 90 Hz with intensities of 0.3 and 0.7 g, we discerned a consistent trend of diminished MTT-based cell viability within 2 days (Figure 3A-C). However, this reduction exhibited variation based on the specific cell line. Notably, a significant decline was evident in the TT2 and U2OS lines, while the MG63 line showed no such reduction. Furthermore, when assessed through a scratch-based cell motility assay, the impact of LIV was significant across all three cell lines (Figure 3D-F).

The decision to apply v-LIV at intensities of 0.3 and 0.7 g was based on the results of the following analyses. In terms of G levels, our findings indicated that among the tested magnitudes (0.1, 0.3, 0.5, and 0.7 g), the 0.7 g level demonstrated the most pronounced impact in terms of suppressing the migration of OS cells (Figure S3). Moreover, concerning the influence of vibration directions, we evaluated the effects of all four variations of vibrations, each applied at an angle, specifically at a frequency of 90 Hz with an intensity of 0.7 g. Among these, v-LIV emerged as the most potent in exerting these effects (Figure S2A-F).

### Alterations in cell shape of MSCs in response to LIV

**Figure 4:**
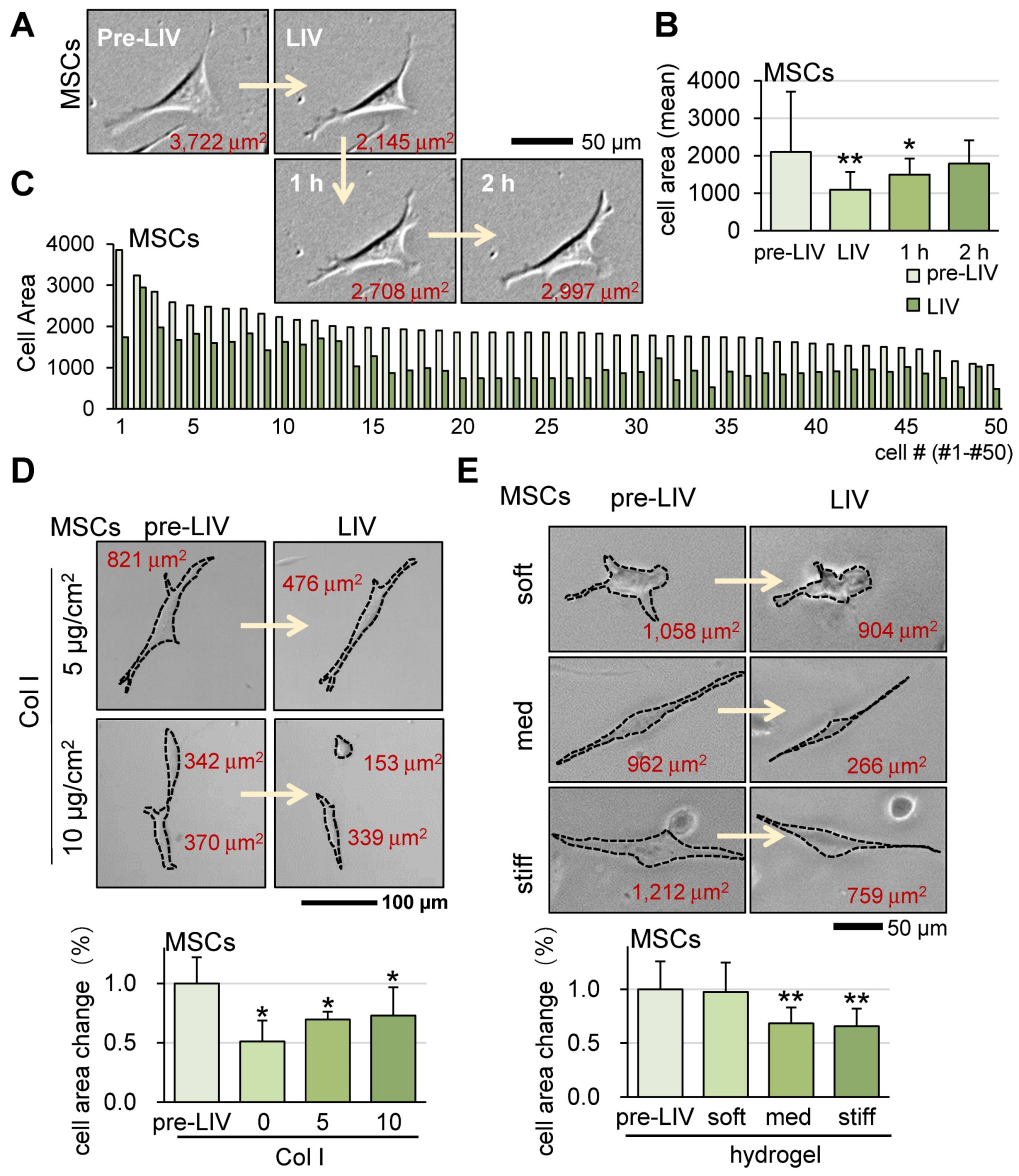
** Alterations in cell shape of MSCs in response to LIV.** LIV was applied vertically (v-LIV) at 90 Hz with a level of 0.7 g. The single and double asterisks indicate p < 0.05 and 0.01, respectively. (A&B) Transient shrinkage of MSCs in response to LIV. (C) Reduction of the surface area for 50 MSCs by LIV. (D) Effects of type I collagen coating on the shrinkage of MSCs in response to LIV. (E) Shrinkage of MSCs in soft, medium, and stiff hydrogels in response to LIV. Of note, storage moduli G' is 1.2 kPa (soft), 4.3 kPa (med), and 7.6 kPa (stiff).

Alongside OS cells, we also investigated the impact of LIV on murine MSCs at a frequency of 90 Hz and an intensity of 0.7 g. Similar to the outcomes observed for OS cells, v-LIV prompted a reduction in the surface contact area of MSCs (Figure 4A-C). Moreover, the presence of a collagen-coated surface and the utilization of a soft hydrogel substrate were found to diminish the responsiveness of MSCs to LIV (Figure 4D-E). We also observed that the same LIV reduced the surface contact area of human bone marrow-derived MSCs (hMSCs) (Figure S4A).

### Suppression of tumorigenic behaviors by LIV-treated MSC CM

**Figure 5:**
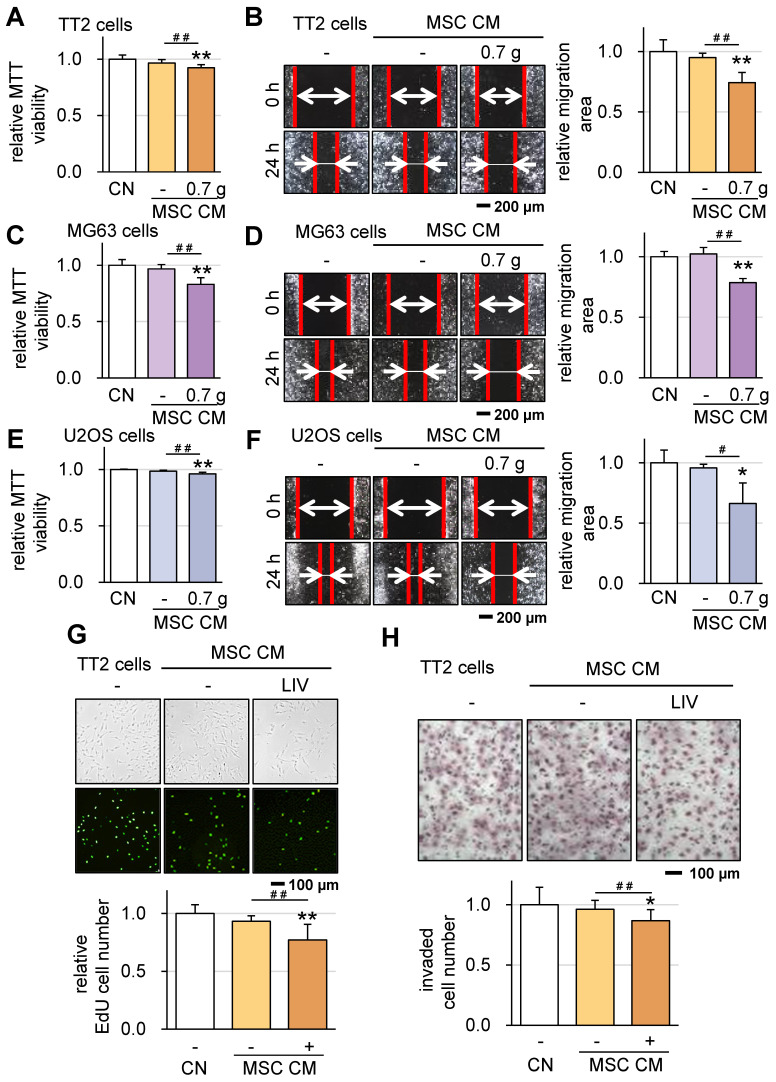
** Suppression of tumorigenic behaviors of OS cells in response to LIV-treated MSC conditioned medium (CM).** LIV was applied vertically (v-LIV) at 90 Hz with a level of 0.7 g. The single and double asterisks indicate p < 0.05 and 0.01, respectively. CN = control, and CM = conditioned medium. (A&B) Reduction in MTT-based viability and scratch-based motility of TT2 OS cells in response to LIV-treated MSC CM. (C&D) Reduction in MTT-based viability and scratch-based motility of MG63 OS cells in response to LIV-treated MSC CM. (E&F) Reduction in MTT-based viability and scratch-based motility of U2OS OS cells in response to LIV-treated MSC CM. (G) Reduction of EdU-based proliferation of TT2 OS cells in response to LIV-treated MSC CM. (H) Reduction of transwell invasion of TT2 OS cells in response to LIV-treated MSC CM.

In line with our previous findings 16, we have successfully generated iTSCs from MSCs. Building on this, we explored the feasibility of generating iTSCs from MSCs exposed to LIV. This involved the application of LIV to MSCs daily, administered twice for 20-minute intervals with a 3-hour interval in between. After 2 days of LIV exposure, CM was collected. Our results revealed a significant outcome: while CM derived from MSCs alone did not exhibit anti-tumor properties, LIV-treated MSC-derived CM displayed notable OS-suppressive abilities. Specifically, the application of LIV at a frequency of 90 Hz with an intensity of 0.7 g led to reduced MTT-based viability and diminished scratch-based motility for TT2 cells (Figure 5A-B), MG63 cells (Figure 5C-D), and U2OS cells (Figure 5E-F). A decreased scratch-based motility of OS cells was also observed in LIV-treated hMSC-derived CM (Figure S4B-D). In addition, the LIV-treated MSC-derived CM reduced EdU-based proliferation and inhibited cell invasion, as demonstrated by the transwell-based cell invasion assay, specifically in the case of TT2 cells (Figure 5G-H).

### Tumor-suppressive effects of v-LIV on transcriptomes and cytokine expression

**Figure 6:**
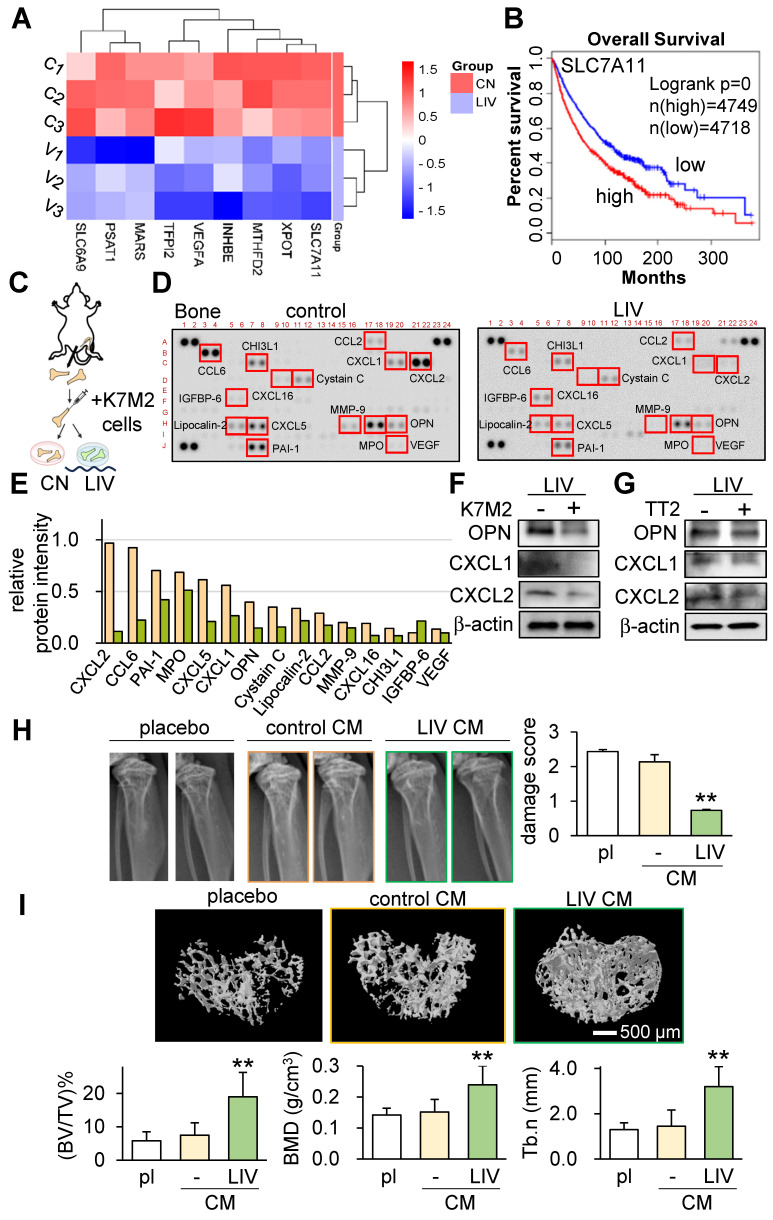
** Tumor-suppressive effects of v-LIV on transcriptome, cytokine expression, and bone homeostasis.** (A) Heatmap of the nine transcripts in the six samples (three control samples such as C1, C2, and C3, and three LIV samples such as V1, V2, and V3), which were significantly altered in TT2 OS cells in response to LIV. Vertical LIV was applied at 90 Hz with a level of 0.7 g, twice for 20 min with a 3-h separation. (B) Survival plot for SLC7A11 transcript. Its high level (threshold at 50% median) provides a significantly shorter survival rate in the TCGA database. (C-E) Reduction in multiple cytokines by daily LIV application to *ex vivo* bone samples. A femur was harvested from a mouse and broken into 2 pieces in the middle. K7M2 OS cells were inoculated into a bone cavity, and the bone samples were cultured for 14 days. LIV was applied daily at 90 Hz with a level of 0.7 g, twice for 20 min with a 3-h separation. (F&G) Reduction in OPN (osteopontin), CXCL1, and CXCL2 in K7M2 OS cells and TT2 OS cells, respectively, in response to LIV at 90 Hz with a level of 0.7 g, twice for 20 min with a 3-h separation. (H) X-ray images of the proximal tibia of C57BL/6 mice, 2 weeks after the inoculation of K7M2 OS cells. The bone damage score was reduced by the administration of LIV-treated CM (n = 10 to 12 tibiae). (I) MicroCT images of the cross-section of the proximal tibia. The image analysis revealed an increase in BV/TV (bone volume ratio), BMD (bone mineral density), and Tb.n (trabecular number) by the administration of LIV-treated CM (n = 10 to 12 tibiae).

Our investigations thus far have underscored the role of LIV in curbing the progression of OS. This is achieved by attenuating oncogenic behaviors in OS cells and concurrently enhancing the anti-tumor attributes within MSCs. In pursuit of comprehending the regulatory mechanisms behind LIV's action, we performed RNA sequencing using TT2 cells, both with and without LIV treatment. The outcomes revealed significant reductions in the transcript levels of several genes, such as SLC7A11, XPOT, MTHFD2, INHBE, VEGFA, TFPI2, MARS, PSAT1, and SLC6A9, as a consequence of LIV exposure (Figure 6A). Intriguingly, according to the TCGA database, elevated transcript levels of these genes (with a threshold set at 50% median) correlated with markedly reduced survival rates (Figure 6B and Figure S5).

To delve into the effects of LIV within the bone-tumor microenvironment, we progressed to an *ex vivo* bone assay. In this context, we harvested a pair of femora from a healthy female mouse and subsequently introduced K7M2 OS cells into the bone marrow cavity.

Our evaluation encompassed the analysis of 111 cytokines in bone samples, with and without the daily application of LIV for 2 weeks. The results strikingly demonstrated the substantial reduction in levels of multiple chemokine ligands (CXCL1, CXCL2, CXCL5, CXCL16, CCL2, CCL6) as well as osteopontin (OPN) in the bone samples subjected to LIV (Figure 6C-F). Concurrently, we also detected a decline in CXCL1, CXCL2, and OPN within TT2 OS cells in response to LIV (Figure 6G).

We next tested the bone-protective effect of LIV-treated CM using the tumor-inoculated tibia of C57BL/6 mice. The three animal groups were the placebo group, the MSC CM treated group, and the LIV-MSC CM treatment group. Mice (~8 weeks old, n = 5 - 6) were inoculated with K7M2 OS cells in their proximal tibia via intratibial injection, and they subsequently received daily tail vein injections of the vehicle, MSC control CM, or LIV-treated MSC CM for 14 days. X-ray images showed that LIV-MSC CM-treated mice exhibited the least significant bone damage in the proximal tibia (Figure 6H). Compared to the X-ray images of the LIV CM group, the images of the placebo and control CM groups showed irregular density patterns or blurred areas, indicating possible bone loss or osteolysis. Additionally, analysis of micro-CT images in the proximal tibia showed that bone volume ratio (BV/TV), bone mineral density (BMD), and trabecular number (Tb.n) significantly increased by the administration of LIV-treated MSC CM (Figure 6I). Collectively, these findings highlight the tumor-suppressive effects of LIV within the intricate milieu of the bone-tumor microenvironment.

### Generation of tumor-suppressive, anti-resorptive CM by VCL overexpression

VCL is a pivotal force-bearing protein within focal adhesions, introducing a potential factor that could influence responses to LIV. Compared to CM derived from control MSCs, our observations underscored that CM sourced from VCL-overexpressing MSCs exhibited notably augmented anti-tumor capabilities.

**Figure 7:**
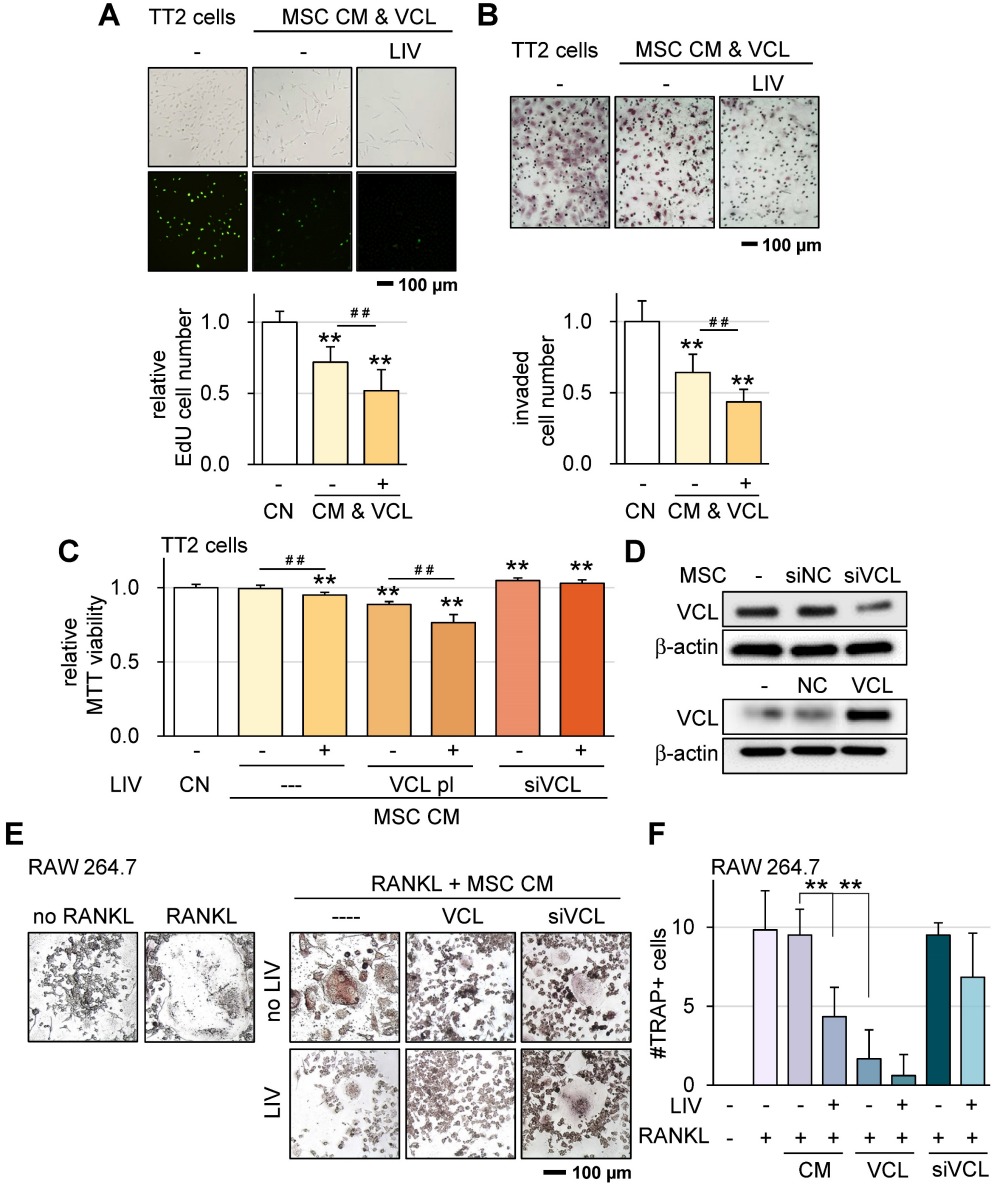
**VCL overexpression promotes MSC CM tumor suppression and suppresses osteoclast differentiation.** LIV was applied vertically (v-LIV) at 90 Hz with a level of 0.7 g. The double asterisks indicate p < 0.01. VCL = vinculin, CN = control, CM = conditioned medium, pl = plasmid transfection, and si = siRNA. (A) EdU-based reduction of TT2 OS cell proliferation by MSC CM in response to LIV and VCL overexpression. (B) Transwell-based reduction of TT2 OS cell invasion by MSC CM in response to LIV and VCL overexpression. (C&D) Alterations in MTT-based viability of TT2 OS cells by MSC CM in response to LIV and over & under-expression of VCL. (E&F) Alteration in multi-nucleated RANKL-stimulated osteoclasts in response to LIV and over & under-expression of VCL.

Upon applying v-LIV at a frequency of 90 Hz with an intensity of 0.7 g to control MSCs, the resulting CM showcased a significant reduction in EdU-based proliferation and inhibited transwell-based invasion. Remarkably, the overexpression of VCL further intensified the tumor-suppressive effects elicited by CM (Figure 7A-B; Figure S6A-D). Moreover, within an MTT-based cell viability assay framework, VCL overexpression emerged as an enhancer of the anti-tumor potential inherent to LIV-treated MSC CM. Consistently, the partial attenuation of VCL expression diminished the inhibitory impact regardless of the application of LIV (Figure 7C-D). The enhanced tumor suppressive effect by VCL overexpression was also observed using hMSCs (Figure S6E-F).

Osteoclasts play a pivotal role in the degradation of bone in the context of OS. Apart from their role in impeding the progression of OS cells, we investigated the impacts of both LIV and VCL overexpression on the maturation of pre-osteoclasts. The outcomes demonstrated that the introduction of LIV-treated MSC-CM significantly impeded the differentiation of RANKL-stimulated osteoclasts, leading to a reduction in the count of multinucleated TRAP-positive osteoclasts (with more than 3 nuclei) (Figure 7E-F). Additionally, the overexpression of VCL further heightened the anti-resorptive efficacy of LIV-treated CM. Conversely, the partial suppression of VCL expression abated the inhibitory effects exerted by MSC CM.

### Enhancement of tumor-suppressive capability of LIV-treated MSC CM by VCL

**Figure 8:**
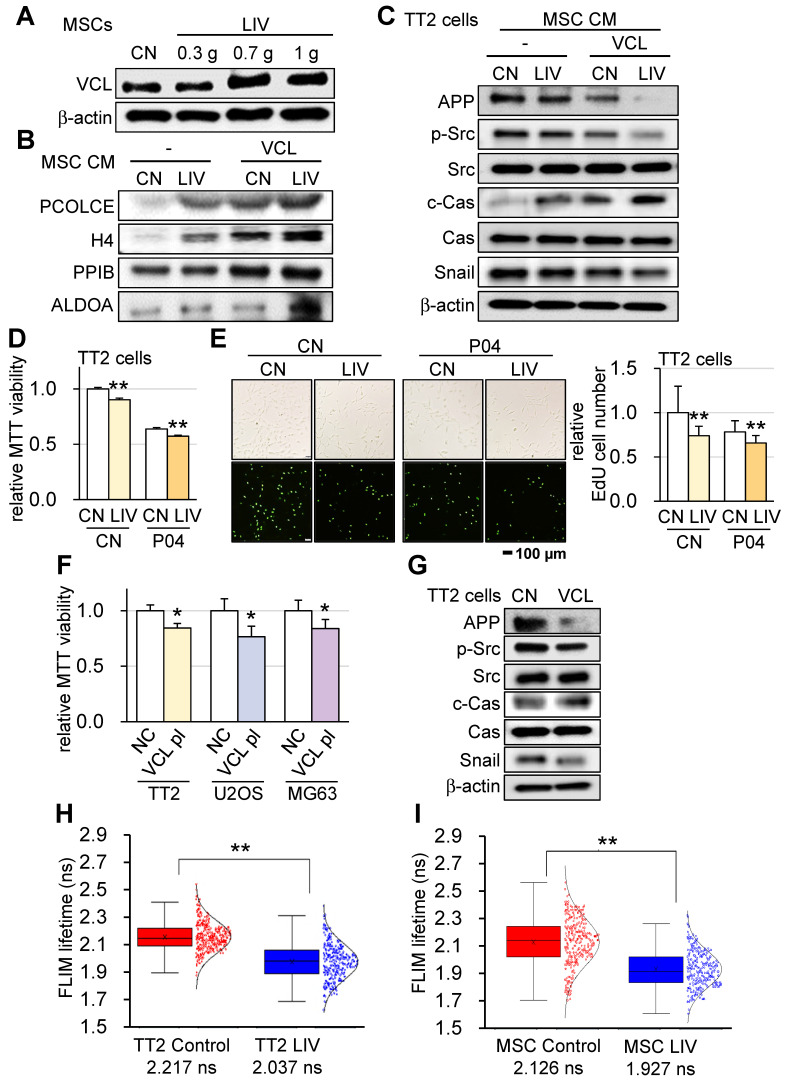
**Enhancement of tumor-suppressing capability of MSC CM by LIV and VCL.** v**-**LIV was applied with a level of 0.3, 0.7, and 1 g. CN = control, CM = conditioned medium, and VCL = vinculin. (A) Elevation of VCL in LIV-treated MSCs. (B) Elevation of tumor-suppressing proteins in the extracellular domain, including PCOLCE, H4, PPIB, and ALDOA in MSC CM by LIV and the overexpression of VCL. (C) Decrease in APP, p-Src, and Snail and an increase in c-Cas in TT2 cells in response to LIV-treated VCL-overexpressing MSC CM. (D) decrease in the MTT-based cell viability of TT2 OS cells in response to LIV-treated MSC CM and 25 µg/ml of P04. (E) Decrease in the EdU-based proliferation of TT2 OS cells in response to LIV-treated MSC CM and 25 µg/ml of P04. (F) Decrease in the MTT-based cell viability of OS cell lines (TT2, U2OS, and MG63 cells) by the overexpression of VCL. (G) Decrease in APP, p-Src, and Snail, and an increase in c-Cas in VCL-overexpressing TT2 cells. (H&I) Reduction in VCL-mediated molecular force of TT2 OS cells and MSCs, respectively, in response to LIV at 0.7 g. The reduction in FLIM (fluorescence lifetime imaging) time indicates a quicker loss of fluorescence signal by a weaker tension in the VCL biosensor.

To elucidate the mechanism underlying the VCL-driven augmentation of tumor-suppressive capabilities, we explored the impact of v-LIV on the expression levels of VCL within MSCs. Interestingly, our findings revealed a significant increase in VCL expression as a result of v-LIV, with the most pronounced elevation observed when the acceleration reached 0.7 g (Figure 8A). Subsequently, we delved into the composition of MSC-CM treated with v-LIV at 0.7 g. This enriched CM exhibited heightened levels of PCOLCE, H4, PPIB, and ALDOA. Notably, these levels experienced a further elevation in response to the overexpression of VCL (Figure 8B). It is noteworthy that the tumor-suppressive properties of these proteins have been reported in our earlier studies 15-17. In line with these observations, both the administration of MSC CM induced by LIV and the CM overexpressing VCL led to the downregulation of Amyloid precursor protein (APP), phosphorylated Src (p-Src), and Snail in TT2 cells. Significantly, PCOLCE has been identified as an extracellular tumor-suppressing protein known to bind to APP, thus shedding light on its role in the observed effects 17. Concurrently, there was an upregulation of cleaved caspase 3 (c-Cas), an established apoptosis marker (Figure 8C).

Given that ALDOA was identified as one of the tumor-suppressing proteins in MSC CM, we sought to extend this analysis to evaluate the role of an ALDOA-derived peptide, P04 (IGEHTPSALAIMENANVLAR). The result showed that the MTT-based cell viability and EdU-based proliferation of TT2 cells was significantly reduced by the administration of 25 µg/ml of P04, and the combination of P04 and LIV enhanced its anti-OS ability (Figure 8D-E). We then proceeded to examine the direct effect of VCL on OS cells. The results showed that the overexpression of VCL in OS cell lines (TT2, U2OS, and MG63) significantly reduced the MTT-based cell viability (Figure 8F). Consistently, the overexpression of VCL in TT2 cells decreased APP, p-Src, and Snail, while c-Cas was significantly increased (Figure 8G). Besides TT2 OS cells, the effect was observed with U2OS OS cells (Figure S6G).

### Reduction of VCL-acting force by LIV

Having established that VCL augments the tumor-suppressive potential of MSC-CM, we analyzed the tensile force exerted on VCL with and without the administration of LIV. The results obtained through fluorescence lifetime imaging (FLIM) revealed that implementing v-LIV led to a diminished tension experienced by the VCL biosensor, both within TT2 OS cells and MSCs. This decrease in tension indicated a reduction in the molecular force transmitted via VCL in response to LIV (Figure 8H-I). Importantly, this observation was congruent with the observed decline in cell contact area induced by LIV, signifying a weakening of the attachment between cells and their substrate.

### Combined effects of LIV-treated MSC CM with chemotherapeutic agents

**Figure 9:**
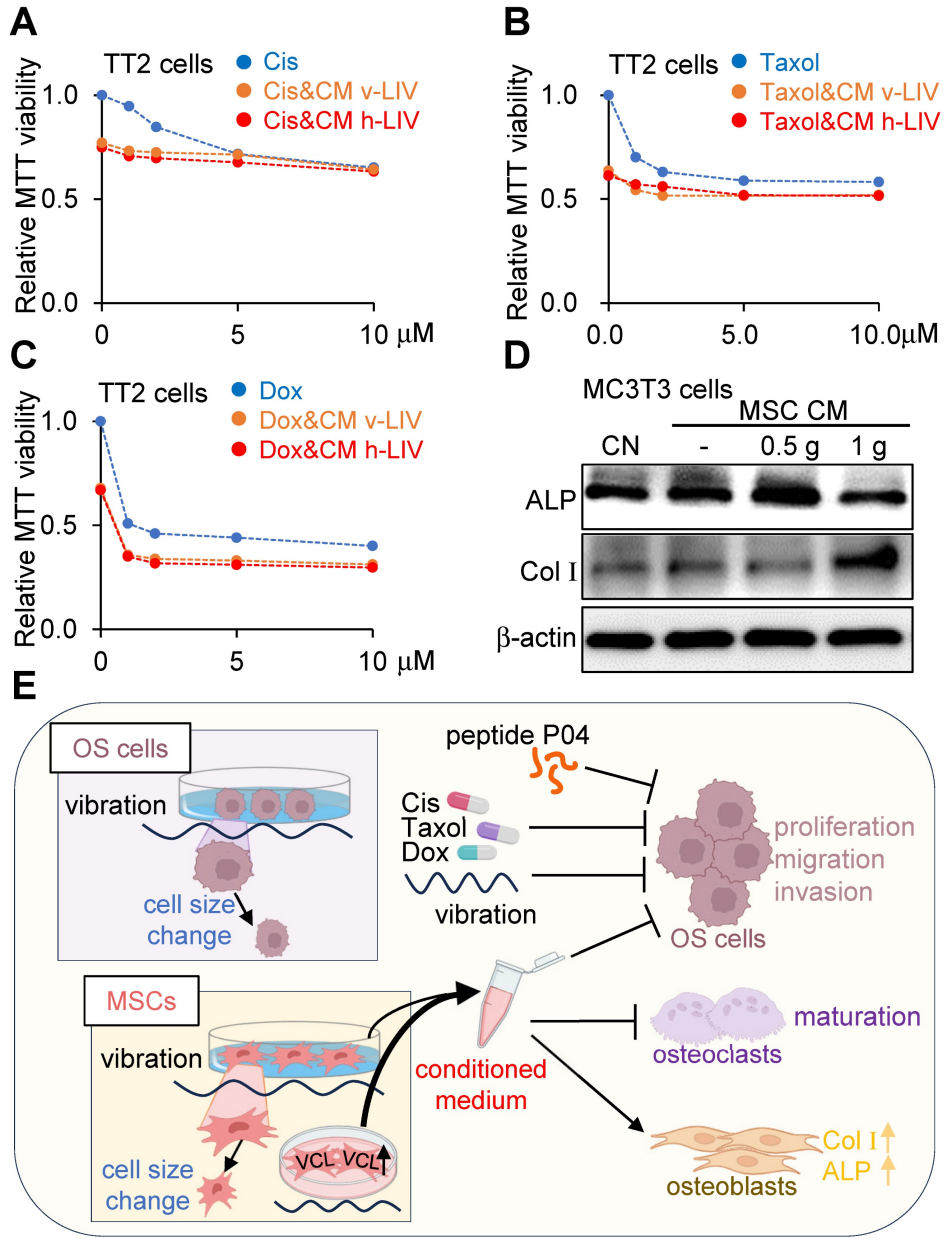
** Combined effects of LIV-treated MSC CM with chemotherapeutic agents, and the proposed mechanism of tumor suppression.** v-LIV or h-LIV was applied at a level of 0.7 g. CN = control, and CM = conditioned medium. Cis = Cisplatin, and Dox = Doxorubicin. (A-C) Combined effects of LIV-treated MSC CM with Cisplatin, Taxol, and Doxorubicin, respectively, on MTT-based viability of TT2 OS cells. (D) Upregulation of ALP (alkaline phosphatase) and Col I (type I collagen) in MC3T3 osteoblasts in response to LIV-treated MSC CM. (E) Proposed mechanism of LIV-driven tumor suppression.

We investigated the impact of concurrent administration of LIV with cisplatin, doxorubicin, and paclitaxel - three prominent chemotherapeutic agents widely employed in clinical anticancer treatments. LIV was applied either in the vertical or horizontal orientation at a magnitude of 0.7 g. The outcomes demonstrated that the combined utilization of LIV-induced MSC CM with the chemotherapeutic agents contributed to reduced MTT-based cell viability of TT2 OS cells (Figure 9A-C). Additionally, when MC3T3 osteoblasts were cultured in LIV CM with applied accelerations of 0.5 g and 1 g, there was an augmentation in osteogenic gene expression, including Col Ι and alkaline phosphatase (ALP) levels (Figure 9D).

## Discussion

This study establishes that LIV exerts a dual effect: inhibiting the progression of OS cells while simultaneously promoting the conversion of MSCs into iTSCs (Figure 9E). Through modal analysis, we anticipated that vibration frequencies ranging from 10 to 100 Hz would effectively provide mechanical stimulation to cultured cells. Moreover, we supplied compelling evidence that LIV at 60 and 90 Hz frequencies, coupled with accelerations of 0.3 to 0.7 g, induced notable morphological transformations in both OS cells and MSCs. Remarkably, CM, harvested from LIV-treated iTSCs at 90 Hz and 0.7 g, not only displayed robust capabilities in suppressing viability, proliferation, motility, and invasive tendencies of OS cells but also showed bone-protective effects in OS-induced mice. Additionally, the upregulation of VCL expression heightened MSCs' responsiveness to LIV, consequently significantly enhancing the efficacy of CM in inhibiting OS cells. Noteworthy tumor suppressor proteins, namely PCOLCE, H4, and ALDOA, previously reported for their roles, were identified in CM derived from LIV-treated MSCs. Furthermore, a noteworthy discovery was identifying the cooperative effects of LIV-treated CM with chemotherapy agents. These findings underscore the viability of LIV-induced generation of tumor suppressor iTSCs and highlight the pivotal role of VCL in priming cells to be receptive to the effects of LIV.

In addition to the direct application of LIV, an unconventional approach involves the formation of iTSCs, wherein tumorigenic signals like the overexpression of β-catenin in the Wnt signaling pathway or administration of activators targeting the Wnt and PI3K pathways are harnessed to counteract tumorigenesis by modulating the proteome 35. The distinct advantages of utilizing CM over conventional chemotherapy drugs are noteworthy. CM, containing many therapeutic proteins, holds the potential for evading inflammatory responses typically associated with allogeneic MSCs. By leveraging LIV to induce the generation of iTSCs, we have advanced the efficacy of the anti-OS effect. While our study primarily employed mouse bone marrow-derived MSCs, we extended our investigations to include human-derived MSCs, demonstrating the capability to generate iTSCs and produce tumor suppressor CMs.

The augmented expression of VCL bolstered the efficacy of LIV-induced iTSCs. Extensive research underscores the pivotal role of VCL in transducing mechanical forces to the actin cytoskeleton 36. Intriguingly, our employment of fluorescence lifetime imaging (FLIM) revealed that v-LIV led to the VCL biosensor within TT2 OS cells and MSCs responding to a decrease in the molecular force transmitted through VCL by LIV. The underlying reasons behind LIV's attenuation of force acting on VCL and the phenomenon of VCL overexpression in MSCs enhancing their LIV-driven anti-tumor potency remain elusive. A plausible explanation is that the heightened expression of VCL diminishes the force exerted on individual VCL molecules, and this reduced molecular force might be pivotal in triggering alterations in cell shape. Changes in cell morphology and adhesion, followed by cytoskeletal reorganization, can regulate signaling pathways, such as those involving focal kinase (FAK) and ERK1/2 37, resulting in the transformation of MSCs into iTSCs. Given that VCL interacts with the focal adhesion complex and actin filaments, further analysis is warranted to unravel the intricate role of VCL in mediating responses to LIV.

This study revealed heightened ALDOA, PCOLCE, H4, and PPIB levels in MSC CM subjected to LIV treatment. Notably, among these transcripts, PCOLCE and H4 are tumorigenic and substantially expressed in OS, as demonstrated by Kexin et al. 17. PCOLCE, investigated by these researchers, emerged as an extracellular tumor suppressor protein. Further investigation highlighted the interaction between PCOLCE and the cell surface protein APP, with elevated APP transcripts correlating with reduced survival in sarcoma patients 17. The elevated c-Cas levels indicate enhanced proteolytic activity, signaling that cancer cells undergo apoptosis. Cancer cells often favor energy production via glycolysis, manifesting the Warburg effect. ALDOA, a pivotal glycolytic enzyme, has been identified as an extracellular tumor suppressor protein in breast cancer 15. In this study, we combined the ALDOA-derived active compound P04 with LIV to synergistically inhibit the viability and proliferation of OS cells.

LIV presents a non-invasive, secure alternative, effectively substituting traditional exercise. Clinical investigations emphasize the bone-density benefits of low-magnitude vibrations. Specifically, vibrations with low amplitude (≤ 1g) and high frequency (≥ 30Hz) are advantageous for compromised bone mass, distinct from healthier bones 38, 39. Across cancer scenarios, animal models exhibit improved bone health due to vibrations, though outcomes diverge based on intensity and frequency. One study showed a linear response from 0.1 g to 0.9 g at 30 Hz 40. Conversely, Lynch et al. found no heightened anabolic effects from 1 g to 0.3 g (90 Hz), enhancing murine bone mineral content 41. Selecting the ideal amplitude and frequency hinges on cell type and intended results. For example, 0.3 g, 90 Hz vibrations notably stimulate osteogenic gene expression in rat bone marrow MSC cells 42. Meanwhile, MLO-Y4 osteocytes exhibit diminished osteoclast-forming RANKL levels at 60 Hz compared to 30 Hz or 90 Hz (0.3 g) 43. Consequently, determining optimal LIV frequency and amplitude, tailored to specific cancer types, requires further exploration.

Reportedly, mechanical forces originating from cell adhesions can transmit to the nucleus, inducing chromatin stretching 44. Consequently, there is a possibility that mechanical signals resulting from LIV may be communicated to the nucleus, influencing gene expression through the cytoskeleton and linker of nucleosleketon and cytoskeleton (LINC) complexes 45, 46. While LIV has the potential to elicit elastic deformation of the nucleus, thereby impacting chromatin accessibility 47-49, conclusive evidence is still lacking regarding whether chromatin remodeling is a cause of the alterations in gene expression 44, 50.

The current study has several limitations. Firstly, the complex nature of the bone microenvironment within the human body involves various bone cell types, the bone matrix, and factors that regulate bone metabolism and blood supply 51, 52. Currently, there needs to be more research investigating the integration of multiple signaling pathways triggered by LIV within this intricate skeletal microenvironment, and the underlying mechanisms of mechanosensory signaling. Secondly, the effectiveness of LIV may vary among patients due to the heterogeneity of osteosarcoma cells, as well as factors like chromosomal abnormalities and mutations. Additionally, most ongoing clinical trials primarily focus on having patients stand or squat on vibration platforms 53-57, highlighting a noticeable gap in localized treatment options.

In conclusion, this study reveals the potential of LIV to simultaneously inhibit the progression of OS cells and facilitate the transformation of MSCs into iTSCs. By harnessing the mechanical stimulation of LIV, we have shown how iTSC-derived CM exhibits potent anti-tumor effects, effectively suppressing OS cell viability, motility, and invasion. Notably, VCL emerges as a critical player, amplifying the responsiveness of MSCs to LIV and enhancing the efficacy of CM. The unique interplay of proteins like PCOLCE, H4, PPIB, and ALDOA within LIV-treated MSC CM underscores their tumor-suppressive attributes. As of now, there are limited treatment options available for OS, particularly in cases of recurrent and metastatic OS. The proposed LIV therapy holds promise in augmenting the tumor-suppressive abilities not only of MSCs but also of various other cell types. The present study, if applied to other types of cells such as T cells, may potentially bolster cell-based cancer treatments like CAR-T cell immunotherapy.

## Supplementary Material

Supplementary figures.

## Abbreviations

ALDOA: Aldolase A
ALP: alkaline phosphatase
ANOVA: a one-way analysis of variance
APP: Amyloid precursor protein
c-Cas: cleaved caspase 3
CM: conditioned medium
Col Ι: type Ι collagen
FLIM: fluorescence lifetime imaging
G: gravitational acceleration
hMSCs: human bone marrow-derived MSCs
h-LIV: horizontal low-intensity vibrations
H4: Histone H4
iTSCs: induced tumor-suppressing cells
OS: Osteosarcoma
LIV: Low-intensity vibration
MSCs: mesenchymal stem cells
PCOLCE: Procollagen C-endopeptidase enhancer
PPIB: Peptidylprolyl isomerase B
p-Src: phosphorylated Src
VCL: Vinculin
v-LIV: vertical low-intensity vibrations
