# Supplementary material for: Enhancing anti-tumor potential: low-intensity vibration suppresses osteosarcoma progression and augments MSCs' tumor-suppressive abilities
Source: Theranostics. 2024 Jan 27;14(4):1430–49. doi: 10.7150/thno.90945 (PMC10879868; doi:10.7150/thno.90945)
Supplement: Supplementary file 1 — Supplementary figures. [file thnov14p1430s1.pdf]

Supplementary information

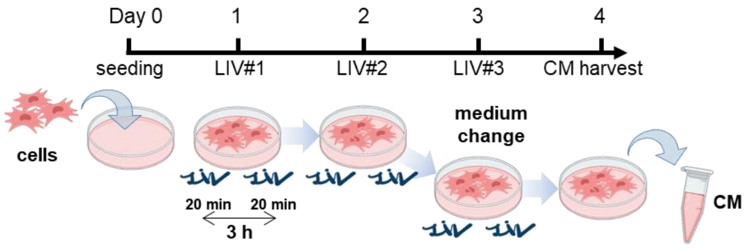

Suppl. Figure 1. The procedure of the application of LIV. After seeding cells on day 0, LIV was applied on days 1, 2, and 3. LIV was 20 min each, and it was applied twice with a 3-h separation. The culture medium was changed fresh before the application of LIV on day 3. The typical LIV condition was at 90 Hz with a 0.7 g-level.

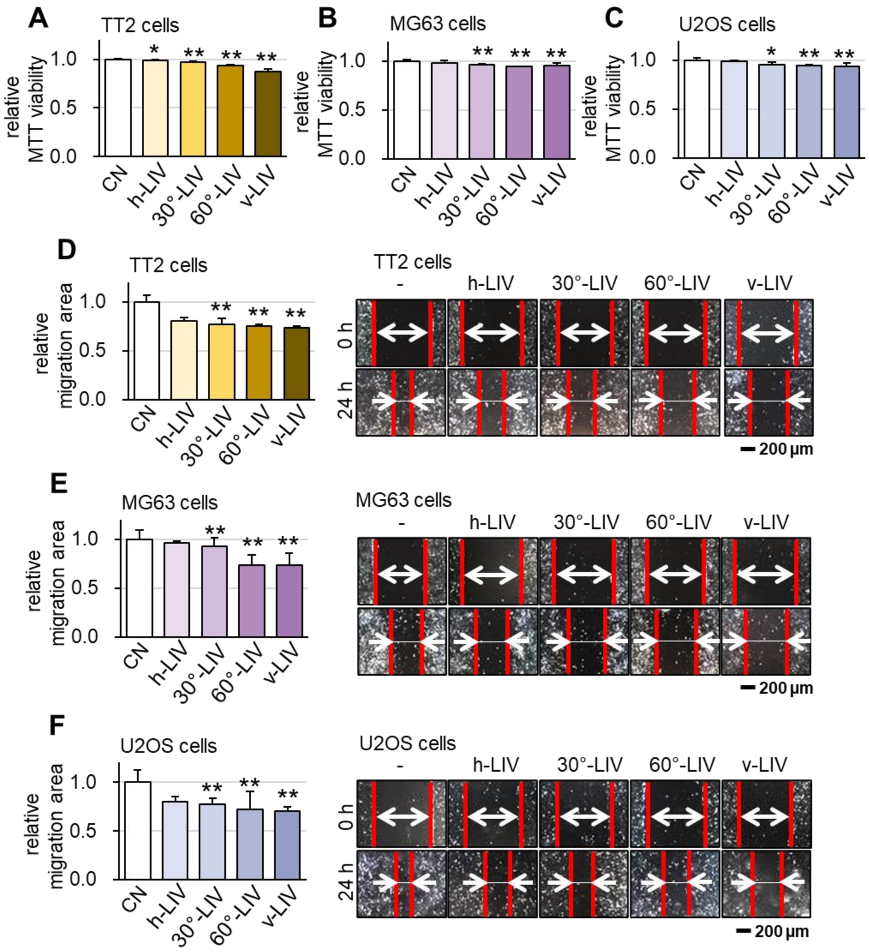

Suppl. Figure 2. **Effect of LIV on the viability and motility of OS cells.** LIV was applied horizontally (h-LIV), 30˚, 60˚, and vertically (v-LIV) at 60 Hz with a level of 0.7 g. The single and double asterisks indicate $p < 0.05$ and 0.01, respectively. (A-C) MTT-based viability of OS cells (TT2, MG63, and U2OS) in response to LIV. (D-F) Reduction in scratch-based motility of OS cells (TT2, MG63, and U2OS) in response to LIV.

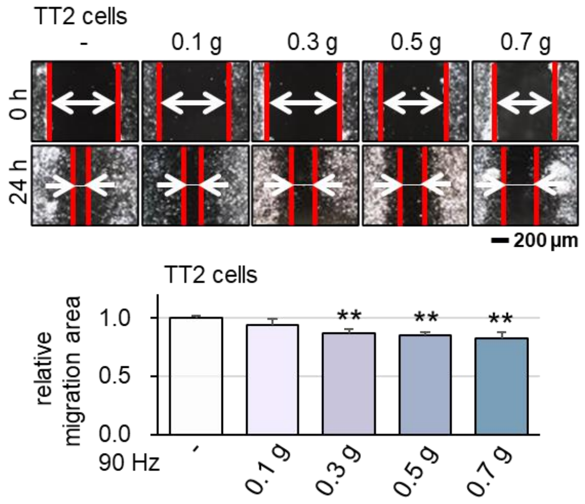

Suppl. Figure 3. **Reduction in the motility of TT2 OS cells in response to LIV.** LIV was applied vertically (v-LIV) at 60 Hz with a level of 0.1, 0.3, 0.5 and 0.7 g. The double asterisks indicate $p < 0.01$.

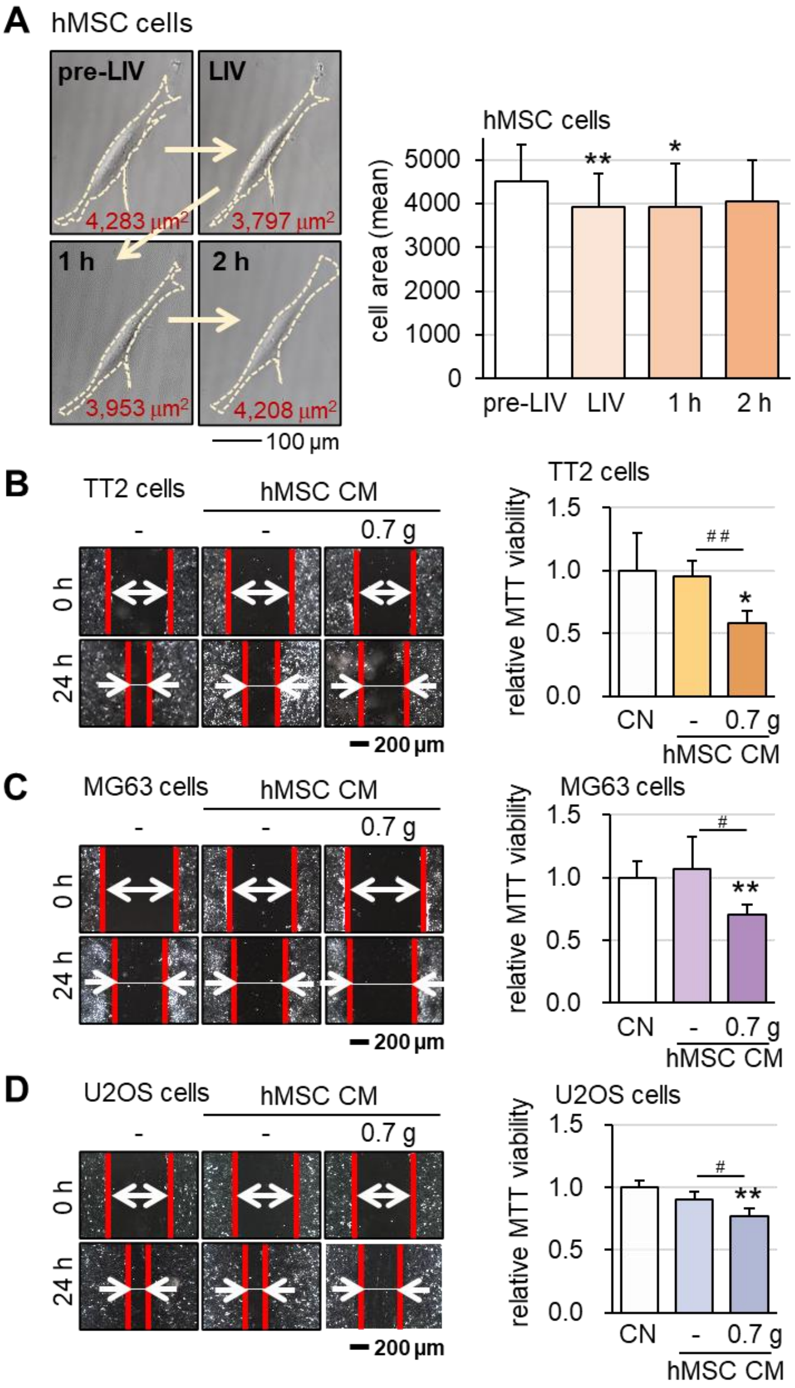

Suppl. Figure 4. **LIV-driven alterations in hMSC cell shape as well as suppression of two-dimensional motility of OS cells in response to LIV-treated hMSC CM.** LIV was applied vertically (v-LIV) for 20 min at 90 Hz with a level of 0.7 g. The single and double asterisks indicate $p < 0.05$ and 0.01, respectively. (A) Transient shrinkage of hMSC cells in response to v-LIV. (B-D) Reduction in scratch-based motility of TT2, MG63, and U2OS cells, respectively, in response to LIV-treated hMSC CM.

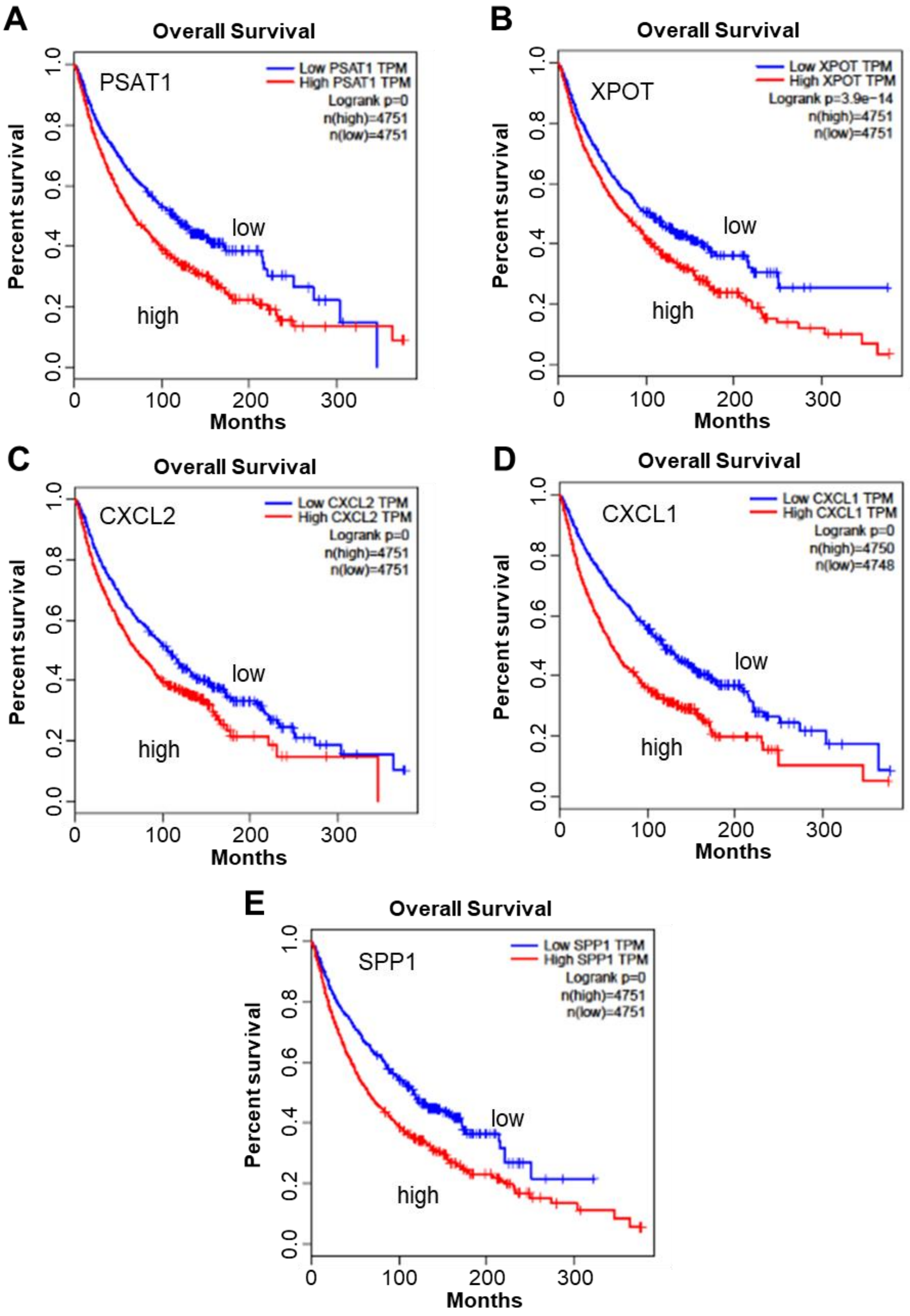

Suppl. Figure 5. **Survival rate plots for the selected transcripts that are responsive to LIV.** (A-E) Survival plots for PSAT1, XPOT, CXCL2, CXCL1, and SPP1 transcripts, respectively. Their high transcript levels (threshold at 50% median) provide a significant reduction in the survival rate in the TCGA database.

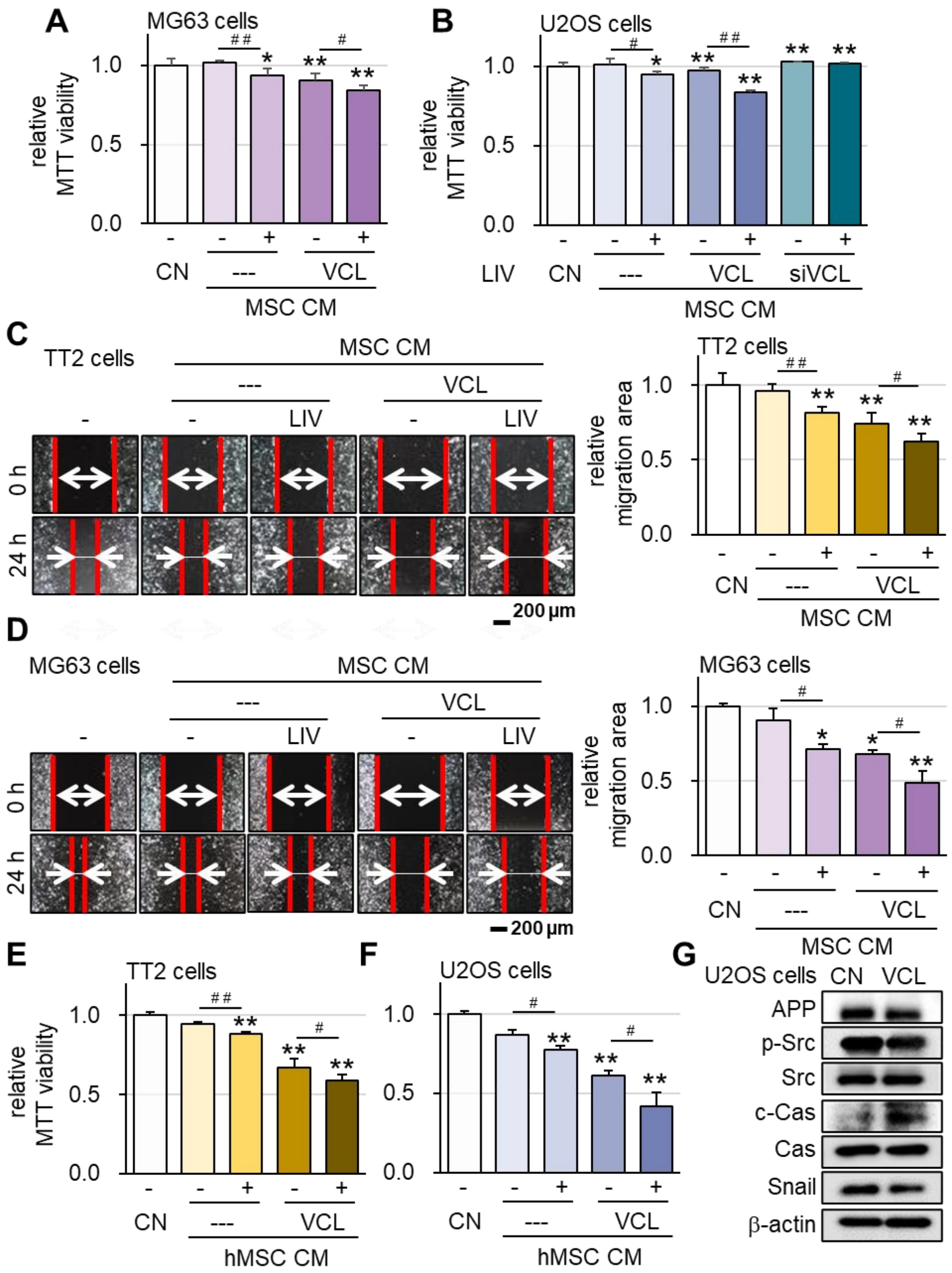

Suppl. Figure 6. **Vinculin overexpression enhances the tumor-suppressive capability of MSC CM.** LIV was applied vertically (v-LIV) at 90 Hz with a level of 0.7 g. The single and double asterisks indicate $p < 0.05$ and 0.01, respectively. VCL = vinculin, CN = control, CM = conditioned medium, pl = plasmid transfection, and si = siRNA. (A-B) Alterations in MTT-based viability of OS cells (MG63 and U2OS) by MSC CM in response to LIV and over- and under-expression of VCL. (C-D) Reduction in scratch-based motility of OS cells (TT2, MG63, and U2OS) by MSC CM in response to LIV and overexpression of VCL. (E&F) Alterations in MTT-based viability of TT2 and U2OS OS cells by hMSC CM in response to LIV and over & under-expression of VCL. (G) Decrease in APP, p-Src, and Snail, and an increase in cleaved caspase 3 (c-Cas) in VCL-overexpressing U2OS cells.
